# A Flexible Carbon Nanotubes-Based Auxetic Sponge Electrode for Strain Sensors

**DOI:** 10.3390/nano10122365

**Published:** 2020-11-27

**Authors:** Francesco La Malfa, Salvatore Puce, Francesco Rizzi, Massimo De Vittorio

**Affiliations:** 1 Center for Biomolecular Nanotechnologies, Istituto Italiano di Tecnologia (IIT-CBN), Via Barsanti 14, 73010 Arnesano (Lecce), Italy; francesco.lamalfa@iit.it (F.L.M.); salvatore.puce@iit.it (S.P.); massimo.devittorio@iit.it (M.D.V.); 2 Dipartimento di Ingegneria dell’Innovazione, Università del Salento, 73100 Lecce, Italy

**Keywords:** auxetic, foam, porous materials, polyurethane, polydimethylsiloxane, strain gauges, wearable

## Abstract

Soft compliant strain gauges are key devices for wearable applications such as body health sensor systems, exoskeletons, or robotics. Other than traditional piezoresistive materials, such as metals and doped semiconductors placed on strain-sensitive microsystems, a class of soft porous materials with exotic mechanical properties, called auxetics, can be employed in strain gauges in order to boost their performance and add functionalities. For strain electronic read-outs, their polymeric structure needs to be made conductive. Herein, we present the fabrication process of an auxetic electrode based on a polymeric nanocomposite. A multiwalled carbon nanotube/polydimethylsiloxane (MWCNT/PDMS) is fabricated on an open-cell polyurethane (PU) auxetic foam and its effective usability as an electrode for strain-gauge sensors is assessed.

## 1. Introduction

In the last few decades, auxetic materials have attracted higher interest because of their unique properties: they are characterized by having a negative Poisson’s ratio [1,2,3,4], defined as the ratio between transverse strain to longitudinal strain. Auxetic properties are scale-independent; therefore, the property of having a negative Poisson’s ratio is valid from the molecular to the macroscopic scale [5,6]. The first persons to give materials with a negative Poisson’s ratio the name “auxetics” were K. Evans et al. [7], who studied molecular structures with negative Poisson ratio values, based on the model presented by R.F. Almgren [8]. There are many examples of auxetic structures: metals, foams, and polymers to name a few. R. Baughman et al. [9] showed that more than 65% of metals exhibit auxetic behavior along certain crystallographic directions, making them suitable for piezoelectric applications. R. Lakes [10] reported the first case of the fabrication of an isotropic foam with a negative Poisson ratio made of open-cell polyester with re-entrant structures. At the same time, K.W. Wojciechowski [11] solved the first isotropic and thermodynamically stable molecular model of a bulk material showing a negative Poisson’s ratio. There are also examples of auxetic composites: A. A. Pozniak et al. showed that a negative Poisson ratio can be achieved in composites when elliptical structures are included [12].

Due to auxetic properties, remarkable improvements can be found in shear modulus, indentation resistance, energy absorption, fracture toughness, wave propagation, vibration transmission and sound absorption, and synclasticity [13]. All of these enhancements can be obtained in sponge materials if the elementary cell of the auxetic foam material is well-designed: the goal is to have numerous hinge-like cell structures, allowing auxetic materials to expand in a transversal direction when stretched, and to narrow when compression is applied [14]. The potential applications of auxetics are countless: in the textile industry, because of their better energy absorption, improvements in the comfort of clothing are obtained by reducing pressure on the skin; the wear resistance and also breathability of the apparel are enhanced [15,16]. For example, L. Zhou et al. [17] presented an auxetic textile material combined with conventional polyurethane foam with enhanced properties, exploitable in the field of impact protection. In military applications, auxetic composite panels were used to improve the blast-resistance of armored vehicles and protective structures. In aerospace and automotive engineering, auxetics are exploited for their capacities to resist shocks and absorb vibrations by the manufacturing of different parts of the vehicles [18]. In biomedical fields, they are used for prostheses but also bandages, soakable with drugs, that are released once stretching is applied. One example in this field is given by auxetic stents exploited in angioplasty procedures: with respect to regular stents, they can be shrunk and extended in a better way, and possess greater resistance to pressure and twisting moments [18,19]. Artificial bones or robotic exoskeletons are being designed with auxetic geometries, providing structures with optimized mechanical properties, stiffness, and flexibility [20].

Auxetic materials can also be fundamental for creating enhanced actuators and sensors for soft systems and robots or wearable devices. Among the different applications, strain gauge measurements represent one of the main methods for the experimental analysis of mechanical loads in the field of transducer technologies. They exploit the variation of the electrical resistance or electrical capacitance for determining how much strain is applied to a point. Measurements are obtained by the conversion of parameters such as force, pressure, tension and weight into an evaluable change in the measured resistance or capacitance [21].

For obtaining capacitive or resistive wearable strain gauge sensors, flexible and stretchable materials are needed and, due to their negative Poisson’s ratio, auxetic structures allow the material to behave differently with respect to conventional ones. In the case of a longitudinally stressed capacitor filled with an auxetic dielectric, the material reacts by expanding and the distance between the two plates of the capacitor increases; this provokes a decrease in the capacitance value. When normal stress is applied to the plates, the thickness between the plates decreases and the value of the capacitance increases. This behavior can be exploited in wearable applications: for example, in capacitive gloves, where the presence of auxetics is useful for distinguishing two mechanical interactions, such as pinching and touching. In a standard dielectric, for both actions, the dielectric width reduces and the capacitance increases; in the case of an auxetic glove, the touch increases the value of the capacitance, and the pinch decreases it [22]. Similarly, conductive polymeric composites have been exploited in strain sensors. The combination of both electrical and mechanical properties allows a polymer to have better properties, combining high stretchability and durability with electrical conduction in order to change the resistance of a flexible polymer-based strain gauge. In particular, the reasonable cost, the ease of fabrication and the excellent electrical properties of carbon nanofibers and nanotubes make these kinds of conductive composites [23,24,25] a suitable choice to be used as polymeric electrodes. Polymer matrices usually show poorer conductivity values with respect to carbon-based materials. However, adding a very low concentration of the latter into polymer matrices can increase their conductivity. It was shown that, in polymer composites, the main mechanism for electrical conductivity is electron hopping, in which electrons jump between the conductive filling material through a tunneling effect [26,27]. This mechanism is enough to guarantee enhanced electrical properties for composites to execute strain measurements.

Among carbon-based filling materials, conductive carbon nanotubes (CNT) present many advantages for the realization of polymeric soft electrodes in flexible piezoresistive devices: conductivity, high aspect ratio, easy and low-cost fabrication methods, high mechanical strength and repeatability are some of them [28,29]. A feature that has attracted attention in polymer-based piezoresistive sensors is the presence of 3D porous structures coated with conductive nanomaterials. Porous polymer, in fact, has greater properties of deformability than non-porous ones; the dispersion properties of carbon nanotubes improve the electrical conductivity of the composite. F. Scarpa et al. [30] investigated and confirmed the auxetic behavior on systems based on single wall CNT (SWCNT). A stretchable supercapacitor electrode was fabricated with porous graphene and carbon nanotubes for energy storage purposes [31]. R. Iglio et al. [32] presented a strain–pressure sensor based on the decoration of PDMS foam with multiwalled carbon nanotubes capable of detecting ultra-small strain and pressure in compression mode. P. Zhang et al. [33] showed a flexible strain sensor based on porous PDMS sprayed with a carbon black/carbon nanotubes composite for the monitoring of human movements and physiological activity. X. Sun et al. [34] presented a flexible tactile sensor based on CNT/PDMS nanocomposites, used for 3D contact force detection with high sensitivity and fast response. X. Zhang et al. [35] fabricated a piezoresistive strain sensor by electrostatic deposition of carbon nanoparticles on commercial polyurethane sponges. All these flexible polymeric-based sensors needed the formulation of bendable electrodes, compatible with the required strain gauge mechanical flexibility.

In this work, we present the fabrication process of a new electrode for an auxetic foam-based resistive strain sensor for applications in robotics and wearable systems. The presence of flexible, conductive electrodes made of multiwalled carbon nanotubes/polydimethylsiloxane (MWCNT/PDMS) on an open-cell polyurethane (PU) auxetic foam guarantees the usability of this material in flexible resistance strain sensors. Electrical analysis was performed on the MWCNT/PDMS composite in order to assess its usability as an electrode. We evaluated the electromechanical properties of conventional and auxetic PU sponges combined with the MWCNT/PDMS composite for strain measurements.

## 2. Materials and Methods 

### 2.1. Materials

Polyurethane sponges (PPI (pores per inch) 10, 20, 60) were purchased from Modulor GmbH (Berlin, Germany). Sylgard® 184 silicone elastomer (PDMS monomer and curing agent) and XIAMETERTM® PMX-200 Silicone Fluid (100 cSt) were purchased from Dow Corning (Midland, MI, USA). Multiwall carbon nanotubes (Purity *>* 90%*,* Outside diameter ≈ 10 nm, CNT layer numbers range between 7 and 9) were obtained from Nanocyl SA (Sambreville, Belgium). Stretchable conductive fabric coated with medical silver coating were obtained from Holland Shielding Systems BV (Dordrecht, The Netherlands). All chemicals, including IPA and other organic solvents, were obtained at high-performance liquid chromatography (HPLC) grades with 99.9% purities from Sigma Aldrich (St. Louis, MO, USA). 

### 2.2. Processing of Materials and Electrodes

An ultrasonic bath (BANDELIN Sonorex Digitec DT 106, Berlin, Germany-Power: 120 W, f = 35 kHz) was used to disperse carbon nanotubes into solvent and into polymer elastomer. A magnetic stirrer (M2-D PRO, ARGO LAB, Carpi, Italy) was used to stir the solution and let the organic solvent evaporate. Modification of conventional PU sponges in auxetic and curing of the conductive polymer took place in a vacuum oven (MEMMERT VO49, Büchenbach, Germany). The imaging of CNTs/PDMS coating was performed by scanning electron microscopy (SEM), with an FEI Helios NanoLab 600i (Thermo Fisher Scientific, Waltham, MA, USA).

#### 2.2.1. Auxetic Sponge Modification

The auxetization process is briefly described here. A common PU sponge can be converted into an auxetic through a thermal compression process that modifies the cells in the honeycomb PU structure in a 3D re-entrant cells topology. Under tension, the re-entrant cells tend to move out and under compression, the structure will bend inward, causing a lateral contraction [3,36,37]. The collapsing of the PU structure is permitted after applying compression by a mold and increasing the temperature up to 160 °C, 20 °C less than the polymer’s softening point. To permanently modify the compressed sponge, the temperature is brought back to room temperature, keeping the deformation. In order for the auxetic process to have a good result, the initial material should have an open-cell structure with a low density: 20 PPI (pores per inch) PU sponges were already identified as the best option. Auxetic PU cylinders have been obtained by cutting 4 and 5 cm diameter disks from 20 PPI conventional PU sheets, to test the effects of different compression directions. When a PU sponge with the same diameter as the mold is used, the compression happens to be purely normal (perpendicular to the circular face). In contrast, when a PU sponge with a bigger diameter is used, the compression is not only normal but also radial (tangent to the circular face). We used a mold in a cylinder shape with a diameter of 4 and 5 cm. Disks were put inside and a piston was placed on top and locked with screws, and we chose the suitable volumetric compression ratio $\left(\frac{V_{f}}{V_{i}}=\frac{1}{3}\right)$. The mold was placed in the oven, heated to 160 °C in 20 min, and kept at this temperature for another 20 min. Then, each cylinder was removed from the mold in order to gently move it, avoiding adhesion of the cell to the walls. Then, it was placed back inside the mold and put in the oven again, where the same treatment was applied (ramp to 160 °C and 20 min at this temperature). At the end of the process, the sample was removed from the mold and cooled down to room temperature.

#### 2.2.2. Fabrication of Conductive MWCNTs/PDMS Composite

**Figure 1:**
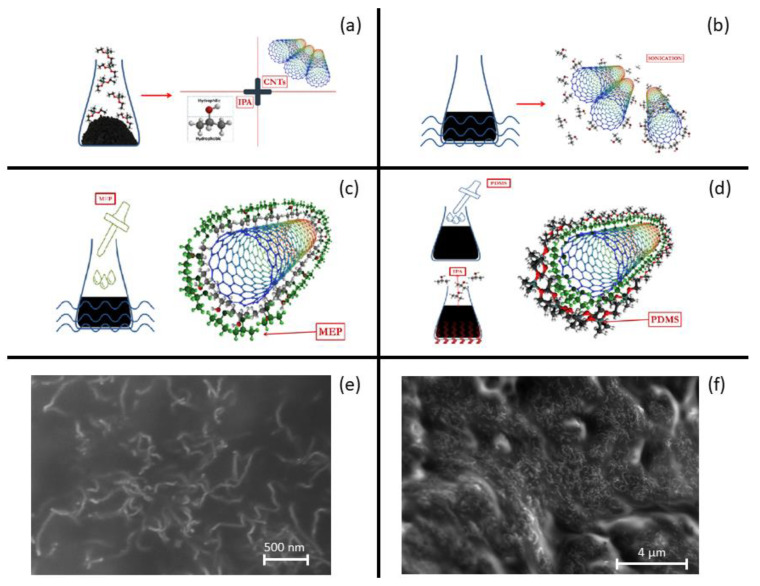
Schematics of CNTs/PDMS hybrid nanocomposite fabrication. (**a**) Detachment and dispersion of aggregated CNT bundles in IPA and (**b**) ultrasonication, (**c**) wrapping of IPA attached CNTs by MEP, (**d**) attachment of PDMS-A to MEP and evaporation of IPA by heating. SEM images of CNTs/PDMS nanocomposite (**e**,**f**).

To obtain a high performance MWCNTs/PDMS device, a homogeneous distribution of MWCNTs must be achieved. This requires the employment of an organic solvent with good dispersion that detaches CNTs and stabilizes the MWCNTs/PDMS solution. Isopropyl alcohol (IPA) was chosen for the partial solubility of CNTs and PDMS in the solvent [38]. CNTs were first dispersed in IPA (100:1 weight ratio). CNT bundles were temporary separated and stabilized by sonication for 30 min; the gaps between them were filled with IPA solvent, forming IPA-coated CNTs. At this point, 20 wt% of methyl group-terminated PDMS (PMX-200) with low viscosity (100 cSt) was added to the IPA-coated CNTs solution and mixed by sonication for 15 min. PMX-200 is a non-volatile polymeric organosilicon material that penetrates the IPA in IPA/CNT complexes and adheres to CNT surfaces. Afterwards, the base agent of PDMS (PDMS-A) was added to the CNT/IPA/MEP solution and mixed by sonication for at least 30 min. Here, the PDMS-A makes direct contact with the MEP phase and surrounds the CNTs. At this point, the IPA component was removed by placing the solution in a hot/magnetic stirrer plate at 50 °C: IPA vaporized slowly and only the composite CNT/MEP/PDMS-A remained. Finally, the curing agent of PDMS (PDMS-B) was added into the composite in a 15:1 weight ratio. Curing in an oven at 80 °C for 1 h completed the process. A cutter was used to achieve a bulk CNT/PDMS cuboid with the dimensions of 30 × 10 × 5 mm. The entire process is shown in Figure 1a–d, while two SEM pictures (Figure 1e,f) demonstrate CNTs uniformity in PDMS.

#### 2.2.3. Electrodes on Auxetic PU with MWCNTs/PDMS Composite

**Figure 2:**
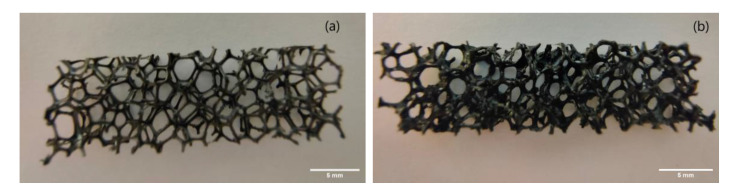
PU cuboid before (**a**) and after coating with CNT/PDMS nanocomposite (**b**). Scale bar: 5 mm.

Conventional and auxetic cube-shaped samples were obtained from PU sponges. Samples of 30 × 10 × 5 mm were obtained by laser cutting and the conductive coating of the PU sponge was evaluated. The sponge functionalization technique was pursued by soaking the sample inside the non-cured CNT/MEP/PDMS mixture for about 30 s. A gentle force was applied by hand while soaking in order to distribute the ink and remove the air inside the holes. Once the soaking finished, the pores of the sponge seemed to be completely filled with mixture, so the pores were opened again with a gentle nitrogen flux and the sample was cured at 80 °C. This process is the building block to obtaining a thin conductive surface layer on the porous PU (see Figure 2).

### 2.3. Mechanical and Electrical Characterization

**Figure 3:**
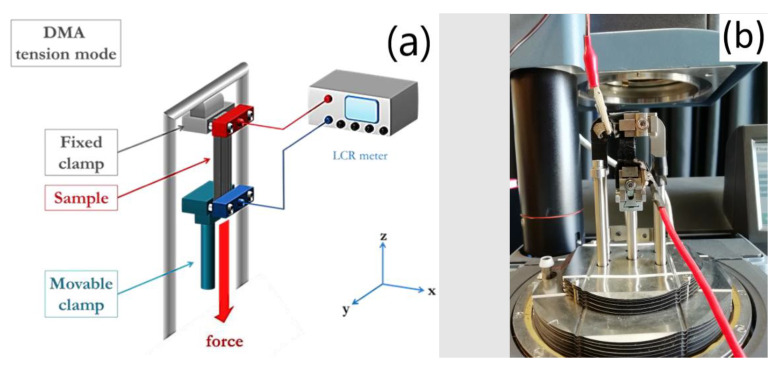
Electrical Dynamic characterization set up (**a**); detail of the set up during the measurement (**b**).

Impedance measurements were carried out with a high precision LCR meter (Keysight E4980A/AL, USA). Tensile strain experiments were carried out with a Dynamic Mechanical Analyzer (DMA Q800, TA Instruments, USA). The combination of the two tools allowed us to perform the experiment with a fine control of the strain in a quasi-static configuration and measure the electrical parameters. In more detail, the DMA was configured in tension mode, in order to control the applied strain, while LCR meter was active, allowing us to simultaneously measure the behavior of electrical resistance (see Figure 3). To monitor the electrical resistance, a high precision multimeter (ISO-TECH-IDM91E) was used. To avoid issues during electrical measurements due to the placement of crocodile clips directly onto the electrode, stretchable conductive fabric was exploited and attached to the electrode through a CNT/MEP/PDMS mixture.

## 3. Results and Discussion

### Electrical and Mechanical Characterization of PU Conductive Sponge

PU/CNT/PDMS sponges were prepared by using two concentrations of CNTs: 2 and 2.5 wt%. These concentrations have been chosen as an average of range boundaries suggested by the CNT supplier and from the literature [39]. The values of conventional and auxetic cuboid samples were compared to the analogous CNT/PDMS sample. As shown in Table 1, because of the different number of conductive paths, electrical resistance is bigger in CNT-coated sponges than in the bulk CNT/PDMS sample. While bulk CNT/PDMS shows a resistance R of ~ 6.6 kΩ for 2 wt% (resistivity ~11 Ω·m) and ~0.9 kΩ for 2.5 wt% (resistivity ~1.5 Ω·m) CNT concentrations, sponge cuboids with a lower concentration of CNT show higher values of R (~60 kΩ for 2 wt% coated sponge and ~28 kΩ for 2.5 wt% one). Therefore, the measured electrical resistance R decreases by increasing the concentration of CNT both in the bulk PDMS blend and PU sponge coating; resistances are up to one order of magnitude less in the CNT/PDMS bulk samples. In the literature, electrical resistance in a conductive polymeric composite saturates to a minimum value for samples containing a CNT wt% higher than 4%, with respect to PDMS weight [40,41].

In comparison to the previous literature, Yogeswaran et al. [28] investigated the electrical behavior of CNT-PDMS nanocomposites, with CNT weight ratio ranging from 3% to 8%. They showed resistivity values from 10^11^ to 1 Ω·m, respectively, suggesting a percolation threshold of the sample at 5 wt%, where an abrupt change starts. This high value is attributed to the CNT coating by polymers. Xu et al. [29] prepared and characterized flexible conductive PDMS composites containing carbon-based fillers. Samples with different concentrations of CNT and carbon fibers (CF) were evaluated. The resistivity value for 1 wt% CNT was 100 Ω·m, while the introduction of CF into the filler improved the value up to 1.8 Ω·m. This improvement was attributed to the interconnection of the CF and CNT in the PDMS matrix. In this respect, the lowest resistivity value obtained in our work (1.5–11 Ω·m) was due to the improved uniformity through the CNT functionalization obtained by MEP introduction. Finally, Iglio et al. [32] presented a PDMS foam decorated with an ink formed by CNTs in ethanol. Concentrations ranging from 7 to 37 mg/cm^3^ gave resistivity values from 70 to 30 Ω·m, respectively. These results are compatible with our foam resistivity ranging between ~100 Ω·m for 2 wt% CNT and ~50 Ω·m for 2.5 wt% CNT.

In order to characterize the electromechanical properties of the functionalized sponge for their exploitation as electrodes in strain gauge sensors, it is really important to assess the elasticity of both conventional and auxetic PU sponges coated with CNT/PDMS. It is necessary to preserve electrical connections inside the composite, especially after stretching; the CNT networks inside the PDMS have to bear continuous deformations. In this work, while electrical parameters were evaluated for concentrations of 2% and 2.5%, for the mechanical analysis, only the samples with CNT wt% of 2% are shown. In fact, with CNT, increasing concentrations result in poorer mechanical properties. It is known that the CNT filler in CNT/PDMS composites may have a negative impact on the regular 3D packing of PDMS molecules. One way to remove this issue is to homogeneously disperse CNTs in order to prevent agglomerations and obtain an efficient load transfer to the filler material; this will enhance both mechanical properties and carrier transport [41], as described in Section 2.2.2.

**Figure 4:**
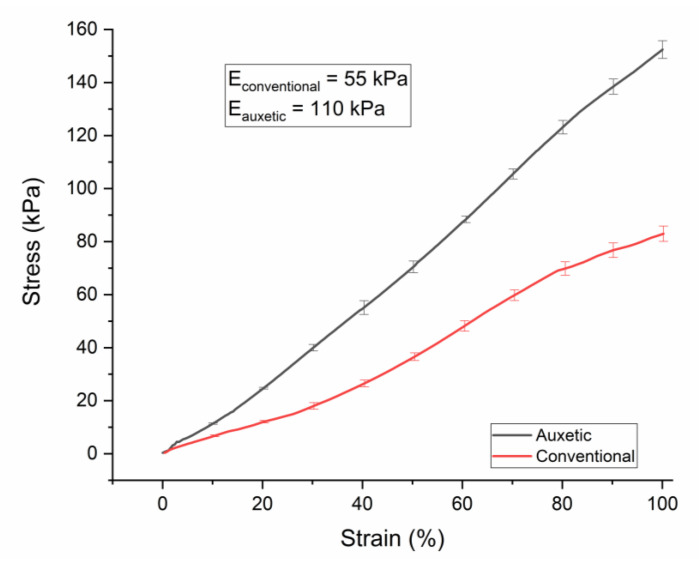
PU conductive conventional and auxetic foam DMA stress–strain responses under tension up to 100%.

For 4 cm diameter conventional and auxetic PU sponges (20 PPI), Young moduli values were measured as 19 and 87 kPa, respectively (see Table 2). After coating with the CNT/PDMS composite, the thickness of the ribs increased, enhancing the values of elastic moduli to 55 kPa for the conventional coated sponge and 110 kPa for the auxetic one (see Figure 4). It is noteworthy that auxetic sponges result as stiffer. We think this is due to the modification of the structure by thermal treatment, although further studies to better assess this feature will be the subject of a forthcoming paper. At strain higher than 30%, the stress–strain curve shows no perfect linear behavior. Therefore, the Young modulus is computed in the linear region of this curve.

**Figure 5:**
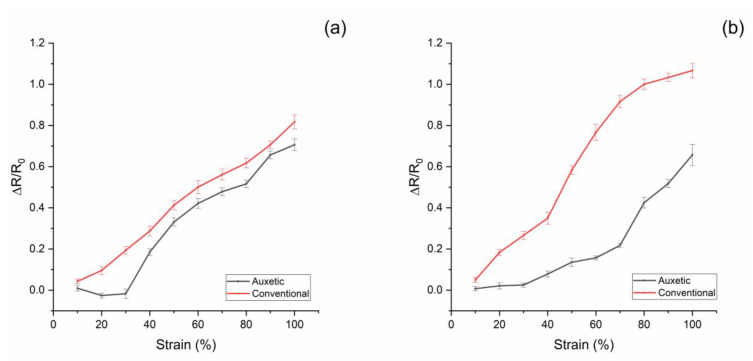
Resistance variation of PU foam samples in CNT/PDMS under tension loading up to 100% (**a**): CNT 2% wt%; (**b**): CNT 2.5% wt%.

For electrical measurements upon strain application, a high precision LCR meter and DMA, exploited in quasi-static configuration, were combined in order to measure the applied strain and the electrical resistance of the conductive sponge. For both conventional and auxetic sponges, at CNT 2 and 2.5 wt%, the resistance increases with the applied strain (see Figure 5).

However, for the auxetic sponge, there is an initial plateau due to the geometric properties of the structure. In fact, the auxetic PU sponge involves the re-entrant structure of cells: when the initial strain is applied, the cell walls, under mechanical stress, return to the standard configuration of the conventional sponge with no expected change to the thickness of the CNT/PDMS coating. From this threshold (strain ~30%), the sample exhibits the conventional behavior, and increasing the strain will cause an increase in the electrical resistance due to the change in the stretched coating [42,43]. In order to define how much the sponge electrode resistance changes with the strain, for both functionalized sponges, conventional and auxetic, the relative change in electrical resistance ΔR/R_0_ to the mechanical nominal strain ε can be measured. This is the slope of the linear region in Figure 4 multiplied by 100. For both auxetic electrodes (2 and 2.5 wt%), it is on average 0.97. In the case of conventional electrodes, for 2 wt%, the slope is 0.87; for the electrode at 2.5 wt%, it is 1.25. In contrast, the auxetic sponge is insensitive to strain of up to 30% in its plateau region. This result suggests an auxetic PU sponge electrode can be exploited in a flexible or wearable sensor without affecting its electric behavior at up to 30% strain. It can be assumed that CNTs’ conductive path could break with the stretching of the structure at strains higher than 30%. In the future, further tests will be performed to study the ageing of the electrode upon multiple stress–strain cycles.

## 4. Conclusions

In conclusion, an electromechanical characterization was carried out for new auxetic electrodes for strain gauge sensors, based on a combination of auxetic process on a PU sponge and the addition of a CNT/PDMS composite. The electrical conductivity of the CNTs/PDMS composite has been characterized. The combination of this composite with the non-conventional mechanical properties of auxetic sponge made in PU has been exploited to functionalize conductive sponges to be used as electrodes in robotics and wearable sensing applications. It provides efficient electrical properties upon stretching and following relaxation. The electrical parameters were measured for PU porous sponges that had been soaked in a conductive CNT/PDMS elastomer composite. The electromechanical properties of auxetic and conventional PU sponges coated with CNT/PDMS have been investigated to assess the elasticity and conductivity of the PU/CNT/PDMS electrodes. Our results showed that an auxetic anisotropic PU sponge functionalized with a CNT/PDMS conductive coating is capable of fulfilling the needs of auxetic flexible electrodes. It is noteworthy that a mechanical threshold for strain has been demonstrated for PU auxetic sponges. It demonstrates an electrical insusceptibility to deformations before 30% strain, a worthwhile property for electric flexible electrodes in flexible strain gauge sensors. In the future, further tests will be done to assess its effective usability in soft robotics and wearable applications, focusing mainly on flexible electrode integration with the sensing element.

## Figures and Tables

**Table 1:** Measured electrical resistance for bulk and PU foam CNT/PDMS.

CNT (wt% PDMS)	Sample	Electrical Resistance R [kΩ]
2%	Bulk CNT/PDMS	6.6
	PU foam in CNT/PDMS	~60
2.5%	Bulk CNT/PDMS	0.9
	PU foam in CNT/PDMS	~28

**Table 2:** Compression and tension stiffness values for PU sponge.

PU Sponge (20 PPI)	Conventional PU [kPa]	Auxetic PU [kPa]
Compression	39	30
Tension	19	87
